# Protective Efficacy of Serially Up-Ranked Subdominant CD8^+^ T Cell Epitopes against Virus Challenges

**DOI:** 10.1371/journal.ppat.1002041

**Published:** 2011-05-19

**Authors:** Eung-Jun Im, Jessie P. Hong, Yaowaluck Roshorm, Anne Bridgeman, Sven Létourneau, Peter Liljeström, Mary Jane Potash, David J. Volsky, Andrew J. McMichael, Tomáš Hanke

**Affiliations:** 1 MRC Human Immunology Unit, Weatherall Institute of Molecular Medicine, University of Oxford, The John Radcliffe Hospital, Oxford, United Kingdom; 2 Department of Microbiology, Tumor and Cell Biology, Karolinska Institutet, Stockholm, Sweden; 3 Molecular Virology Division, St. Luke's Roosevelt Hospital Center, Columbia University Medical Center, New York, New York, United States of America; 4 The Jenner Institute, University of Oxford, Oxford, United Kingdom; NIH/NIAID, United States of America

## Abstract

Immunodominance in T cell responses to complex antigens like viruses is still incompletely understood. Some data indicate that the dominant responses to viruses are not necessarily the most protective, while other data imply that dominant responses are the most important. The issue is of considerable importance to the rational design of vaccines, particularly against variable escaping viruses like human immunodeficiency virus type 1 and hepatitis C virus. Here, we showed that sequential inactivation of dominant epitopes up-ranks the remaining subdominant determinants. Importantly, we demonstrated that subdominant epitopes can induce robust responses and protect against whole viruses if they are allowed at least once in the vaccination regimen to locally or temporally dominate T cell induction. Therefore, refocusing T cell immune responses away from highly variable determinants recognized during natural virus infection towards subdominant, but conserved regions is possible and merits evaluation in humans.

## Author Summary

The body's reaction to infections is dominated by strong immune responses focused on a small number of protein regions, while ignoring the other. These strong responses may be protective against invariant viruses, but fail to control viruses that can change quickly. In particular for the AIDS virus, it happens that the few targeted regions during natural infection are highly variable, and rapidly change and become unrecognized by the mounted responses, i.e. escape. Here, we demonstrated that even the naturally under-recognized protein regions can induce robust responses and confer protection against whole viruses, and therefore should be made use of by vaccination strategies.

## Introduction

In any one individual, CD4^+^ and CD8^+^ T cell responses elicited by virus infection or vaccination focus on a relatively small number of epitopes [1]. This immunodomination results from an interplay of multiple factors, which on one hand determine the abundance of displayed peptide-loaded MHC determinants on the surface of APCs and, on the other hand, affect the numbers of naïve T cells and their capacity to efficiently compete for their cognate determinants and generate effector and memory CD8^+^ T cell populations [2]–[6]. A consequence of immunodominance is that potentially protective responses to a majority of virus-carried determinants might not be used to their full potential or at all by the host immune system.

This issue is particularly pertinent to the human immunodeficiency virus type 1 (HIV-1). Given the huge quasi-speciation of HIV-1 in human populations [7] and its rapid escape from the dominant T cell responses in infected individuals [8]–[11], exploitation of subdominant T cell epitopes by vaccines might stimulate T cell responses against HIV-1 that are more effective than those that occur naturally. The first natural T cell responses to the first-recognized immunodominant epitopes in HIV-1 infection almost invariably lead to rapid selection of escape mutants, while more effective responses to conserved epitopes only arise later [9]. The importance of subdominant epitopes in control of HIV-1 replication *in vivo* was also implied in a study, where HIV-1-infected individuals with a stronger cytotoxic reactivity against several subdominant epitopes had lower virus load than the subjects lacking such activity, while the most immunodominant response against the same epitope in either subjects was identical [12]. Some virus and tumour mouse models have also shown that CD8^+^ T cells specific for subdominant epitopes can contribute to the protective immunity [5], [13]–[17]. Thus, better understanding of the rules governing immunodominance may lead to novel rational strategies for induction of broader, more protective T cell responses against HIV-1 and other viruses.

In preclinical evaluation of HIV vaccines, in the absence of simple functional correlates of protection against HIV-1 [18], various *in vitro* T cell parameters, such as IFN-γ production, are used to evaluate immune responses stimulated by candidate vaccines. However, these assays may not measure the true protective functions. Therefore, we established two surrogate virus challenge models of mice in order to examine how vaccine-stimulated T cell responses protected against a real virus infection *in vivo*. These models employ a replication competent vaccinia virus strain Western Reserve sharing with vaccines the HIV-1-derived immunogen [19], [20], and EcoHIV, which carries a gene coding for envelope gp80 of ecotropic murine leukemia virus in place of the HIV-1 envelope and therefore can efficiently infect mouse cells through receptor called cationic amino acid transporter type 1 [21]–[23]. In this report, we employ these two model challenges for evaluation of the protective efficacies of T cells specific for subdominant HIV-1 CD8^+^ T cell epitopes.

In the experiments reported here, we used the candidate HIV-1 vaccine immunogen HIVA [24], mapped its CD8^+^ T cell epitopes in BALB/c mice and determined their hierarchy [25], [26]. This allowed us to test, by removing more immunodominant epitopes, whether or not cellular immune responses focused on the subdominant determinants could be protective. This question is very important for the HIV-1 vaccine development, because, besides the problem of early virus escape from T cell responses, the enormous variability of the infecting virus could also be tackled by stimulating T cell responses against subdominant epitopes [27] in the most invariant regions of the HIV-1 proteome common to the major clades [28], [29]. We investigated this by removing dominant epitopes from the vaccine and testing whether different vaccine vectors affect the pattern of dominance. We also asked if vaccine administration into anatomically separate sites partially overcomes epitope competition and increases the breadth of elicited CD8^+^ T cell responses. Finally, we assessed the protective efficacy of these up-ranked CD8^+^ T cell responses against virus challenges. The results strongly support further development of the conserved region vaccine strategy and its evaluation in humans.

## Results

### Mapping of CD8^+^ T cell epitopes and their immunodominance hierarchy

**Figure 1:**
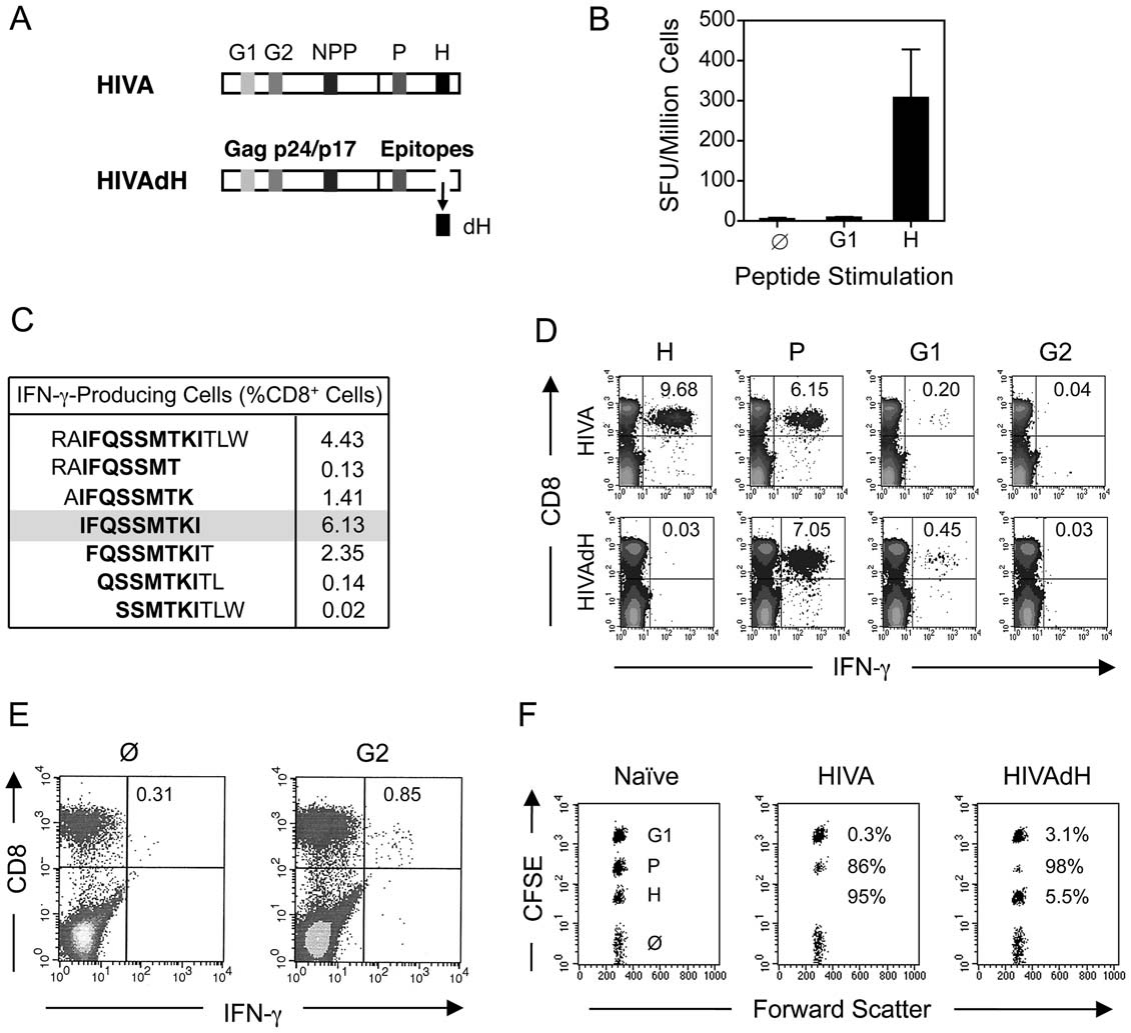
Enhancement of subdominant responses by deletion of immunodominant epitope. A) Schematic representation of the HIVA and HIVAdH immunogens depicting the locations of the H-2^d^-restricted CD8^+^ T cell epitopes and deletion of epitope H. B) Immunogenicity of pTHr.HIVA DNA. A group of 4 BALB/c mice were immunized with a single dose of 100 µg of pTHr.HIVA DNA. Ten d later, splenocytes were isolated and assessed in an *ex vivo* IFN-γ ELISPOT assay using the known G1 and H peptides. Responding cell frequencies are shown as mean±SD. C) Fine mapping of a previously unidentified CD8^+^ T cell epitope P. Groups of 4 BALB/c mice were primed with 50 µg of pTHr.HIVA DNA and boosted with 5×10^6^ PFU of MVA.HIVA 2 wk later. Two wk after the boost, splenocytes were assessed in an ICS assay for IFN-γ production using 14-mer and 6 staggered overlapping 9-mer peptides. D) Immunogenicity of HIVA and HIVAdH immunogens. Mice were immunized with immunogens HIVA or HIVAdH as described in C. Two wk after the boost, splenocytes were tested in an ICS assay for IFN-γ production against the H, P, G1 and G2 epitopes. Inserted numbers indicate IFN-γ producing cells as a percentage of total CD8^+^ splenocytes. E) G2-specific responses are detectable after culture expansion. Splenocytes from immunized mice as in D were pooled and re-stimulated *in vitro* for 5 d with peptide G2 and assessed in an ICS assay for the production of IFN-γ upon G2 peptide restimuation. IFN-γ-producing cells as percentages of total CD8^+^ splenocytes are indicated. F) *In vivo* recognition of subdominant epitopes. Mice were immunized as in D and the cytolytic activity was assessed in an *in vivo* lysis of syngeneic peptide-pulsed cells in naïve and vaccinated animals. Representative examples are shown with inserted numbers indicating the percentages of peptide-specific target killing. Results from one out of four individually immunized and tested animals are shown.

Our research interests lie in development of the T cell component of HIV-1 vaccines stimulating protective CD8^+^ T cell responses. The first-generation T cell immunogen, designated HIVA (Figure 1A) [24], has served as an extremely useful tool for iterative improving of CD8^+^ T cell induction by experimental vaccines in mice, non-human primates and humans [reviewed in [30]]. To increase the power of T cell analysis and study the role of subdominant determinants, H-2^d^ class I-restricted epitopes contained in the HIVA immunogen were mapped in detail. Thus, the most immunodominant epitope of HIVA is RGPGRAFVTI [Env residues 311–320 [31], by us historically designated H for ‘HIV’ [32]] restricted by H-2D^d^ and H-2L^d^ molecules [31], [33]. The H epitope was attached to the C-terminus of HIVA to facilitate quality control of clinical vaccine batches. The H-specific response dominated CD8^+^ T cell responses to other well defined epitopes in HIVA such as H-2K^d^-restricted AMQMLKDTI (Gag residues 197–205, designated G1) and TTSTLQEQI (Gag residues 239–247, designated G2) [34]. Particularly the G1 epitope was previously reported to be immunodominant in BALB/c mice [26], [35], but from HIVA induced hardly detectable responses (Figure 1B and D top). Note that HIV-1 clade A in epitope position 7 contains aspartate rather than glutamate, which is a less immunogenic epitope variant [26]. Using 15-mer peptides overlapping by 11 amino acids (15/11) across the whole HIVA protein [36], a strong CD8^+^ T cell response was detected to the polyepitope region of HIVA in addition to epitope H, which was subsequently narrowed to sequence IFQSSMTKI (Pol 258–266, designated P; Figure 1C). This epitope is not processed from its natural Pol context (E-JI, TH unpublished) due to an adjacent proline immediately upstream of the epitope, which prevents NH_2_-terminal trimming [37]; in that position, HIVA contains alanine. To distinguish impaired G1 epitope processing from other mechanisms of immunodominance, modified immunogen HIVAdH was constructed, from which epitope H was physically removed (Figure 1A), and used to immunize BALB/c mice. While the frequencies of IFN-γ-producing CD8^+^ cells responding to the G1 peptide, and indeed to the P peptide, were noticeably, though not significantly increased, responses to peptide G2 remained in the *ex vivo* ICS assay undetectable (Figure 1D). Nevertheless, G2 responses were primed, because a specific IFN-γ production was detected in HIVAdH-immune splenocytes following a 5-d G2 peptide-driven culture expansion (Figure 1E) in an experimental setting that is highly unlikely to support priming of naïve CD8^+^ T cells. Relative to the HIVA vaccination, increased *in vivo* lysis of G1- and P-pulsed targets was also observed in HIVAdH vaccinated mice (Figure 1F). Thus, this series of experiments demonstrated that a single strong epitope can dominate CD8^+^ T response to vaccination and significantly narrow the specificity of the total response.

### Serial up-ranking of subdominant epitopes by inactivation of dominant epitopes

**Figure 2:**
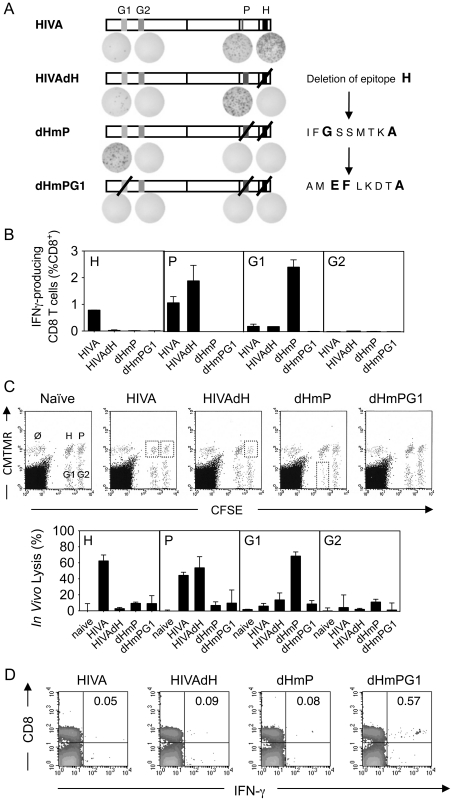
Serial up-ranking of epitopes. A) Groups of 4 BALB/c mice were immunized 2x i.m. with 100 µg of DNA at a 2-wk interval. Two wk later, splenocytes were assessed in an ELISPOT assay for production of IFN-γ upon re-stimulation with peptides indicated above. Shown are schematic diagrams of HIVA and the three modified immunogens with representative examples of the ELISPOT well images. B) Mice were immunized as in A and 4 wk later, splenocytes were assessed in an ICS assay to measure CD8^+^ T cells secreting IFN-γ upon re-stimulation with peptide indicated in the top left corner. Group means±SD are shown. C) Identical groups of mice were assessed for cytolytic activity in an *in vivo* lysis of syngeneic peptide-pulsed cells transferred into naïve or vaccinated animals. Representative examples of dot plots are shown (top) and the peptide-specific lyses are expressed as a group mean±SD (bottom). D) Groups of 3 BALB/c mice were immunized 3x with 100 µg of DNA indicated above the dot plot in 2-wk intervals and the designated NPP peptide-specific responses were assessed by IFN-γ ICS 2 wk later. The FACS plots are representative examples with IFN-γ producing cells as a percentage of CD8^+^ splenocytes shown in the top right panels. All experiments were repeated at least once.

Deletion of immunodominant epitope H had a limited impact on restoring responses to epitopes G1 and G2. To further investigate this phenomenon, we serially inactivated CD8^+^ T cell epitopes in the order of their immunodominance. To maintain the HIVA protein integrity, epitopes P and G1 were mutated rather than deleted by substituting the anchor amino acid residues required for a strong binding to the H-2K^d^ molecule (Figure 2A). The resulting double and triple mutants of the HIVA immunogen were called dHmP and dHmPG1, respectively. Their comparable intracellular expression levels and half-life were demonstrated (Figure S1A), and the altered peptide sequences IFGSSMTKA and AMEFLKDTA were confirmed to be unable to bind H-2K^d^ in a T2 binding assay (Figure S2A). Groups of BALB/c mice were immunized 2x using the modified HIVA immunogens vectored by plasmid DNA and the immune responses were measured in IFN-γ ELISPOT, ICS and *in vivo* killing assays. First, it was confirmed by the absence of IFN-γ production that splenocytes from mice immunized with mutated HIVA vaccines dHmP and dHmPG1 were unable to recognize the altered P and G1 peptides, respectively (Figure 2A and B). Second, consistently in all three assays, immune responses directed against subdominant epitopes were serially recovered, however, not to the same extent. A significant restoration of G1-specific T cell responses, now comparable to those against H and restored P, was observed in dHmP-immunized group, while immunization with dHmPG1 vaccine failed to restore the G2-specific response (Figure 2B and C). Because the response to the G2 epitope remained undetectable even in the dHmPG1-immunized mice, overlapping 15/11 peptides across the whole HIVA immunogen were used again to search for another yet unidentified epitope of a higher rank. Indeed, a response was detected and mapped to a 15-mer peptide NPPIPVGDIYKRWIILGLNK (Figure 2D). This peptide contains a known BABL/c CD4^+^ T helper epitope present in the Los Alamos National Laboratory HIV Sequence Database, and peptide NPPIPVGDI, designated NPP, was shown to be restricted by H-2D^d^ and H-2L^d^ (Figure S2B). Thus, step-wise deletion of immunodominant CD8^+^ T cell epitopes restores responses to the subdominant epitopes in the hierarchy.

### Peptide-MHC affinity, T cell avidity and response kinetics contribute to immunodominance

**Figure 3:**
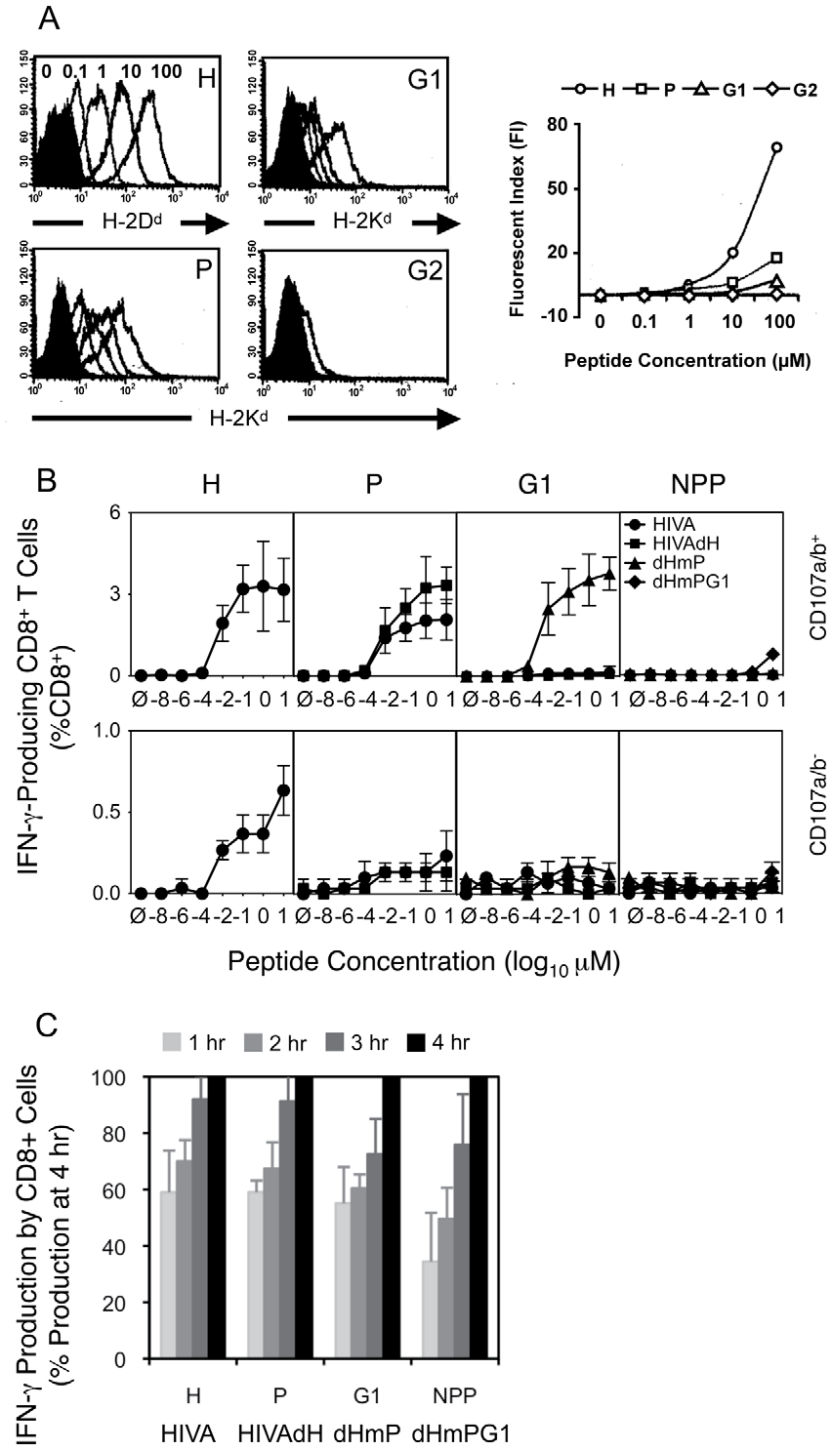
Contributing mechanisms of immunodominance. A) Binding affinities of peptides H, P, G1 and G2 (as indicated in the top right corners) to their respective MHC class I complexes stably transfected into TAP-deficient T2 cell lines were assessed. T2 cell lines were incubated with decreasing peptide concentrations ranging from 100 µM to 0.1 µM indicated above the H peptide histogram and the cell surface peptide-loaded MHC complexes were detected by fluorochrome-conjugated mAb (DB Biosciences) and quantified using FACS flow cytometer. B) and C) Groups of 3 BALB/c mice were given 3x immunizations using 100 µg of DNA expressing the index or mutated forms of HIVA at 3 wk intervals and their T cell responses were analyzed 4 wk later. B) Splenocytes were assessed in an ICS assay using peptides indicated above the graphs at concentrations ranging from 10 to 10^−8^ µM for 5 h. Cells were gated on IFN-γ-producing CD8^+^ T cell populations positive (top) or negative (bottom) for CD107a/b. Note the different y-axis scales on the top and bottom graphs. C) The response kinetics of CD8^+^ T cells re-stimulated with 0.1 µM peptides was assessed in an ICS assay in 1-h intervals for a period of 4 h. Data shown are as mean±SD of [CD8^+^CD107a/b^+^IFN-γ^+^ cells at each time point (%)] ÷ [CD8^+^CD107a/b^+^IFN-γ^+^ at 4 h (%)]. One out of two independent experiments is shown.

In the previous section, we established the hierarchy of T cell responses against HIVA-derived epitopes 10 d after immunization to be H>P>>G1>NPP>>G2. To characterize and compare the corresponding peptides, we assessed the binding affinities of the H, P, G1 and G2 peptides for their respective MHC class I using TAP-deficient T2 cell lines stably transformed with H-2D^d^, H-2K^d^ or H-2L^d^ complexes and found that the peptide binding affinities directly correlated with the immunodominance hierarchy (Figure 3A).

We also measured avidities of T cell responses recovered through the sequential HIVA epitope up-ranking. First, responsiveness of immune splenocytes following 3 plasmid DNA deliveries was measured in an ICS assay upon re-stimulation with peptide concentrations ranging from 10^−8^ to 10 µM for 5 h (Figure 3A). Judging from the curve slopes and *in vitro* peptide concentrations achieving maximum IFN-γ^+^CD107a/b^+^CD8^+^ cell frequencies, 10- to 100-fold less H peptide for HIVA-induced responses was necessary relative to the P peptide for the HIVAdH-induction and G1 peptide for the dHmP-induction. This could in part reflect the predominant binding of H peptide to H-2D^d^, and P and G1 peptides to H-2K^d^. For individual determinants when dominant, the peak cell frequencies at 10 µM peptide concentration were 3.2%, 3.3%, 3.8% and 0.8% of total CD8^+^ splenocytes for peptide H in group HIVA, peptide P in group HIVAdH, peptide G1 in group dHmP and peptide NPP in group dHmPG1, respectively. Thus overall, epitopes H, P and G1 have similar intrinsic immunofgenicities when not dominanted by stronger epitopes. In non-degranulating IFN-γ^+^CD107a/b^−^CD8^+^ cells, only peptide H induced low, but significant IFN-γ response in 0.65% of total CD8^+^ cells following the HIVA vaccination (Figure 3B, bottom).

Finally, a constant 0.1 µM concentration of one of the four H, P, G1 or NPP peptides was used to restimulate immune splenocytes initially for 90 min as a pre-incubation period followed by additional 1 to 4 h (Figure 3C) sufficient for the IFN-γ expression to reach maximum [38]. The time-course suggested a trend whereby the more immunodominant epitopes reached their full IFN-γ/CD107a/b response faster (Figure 3C). Thus, peptide affinity for MHC, T cell avidity and response kinetics are mechanisms contributing to immunodomination of epitopes within HIVA.

### Spatial and/or temporal dominance is required for efficient induction of subdominant responses

**Figure 4:**
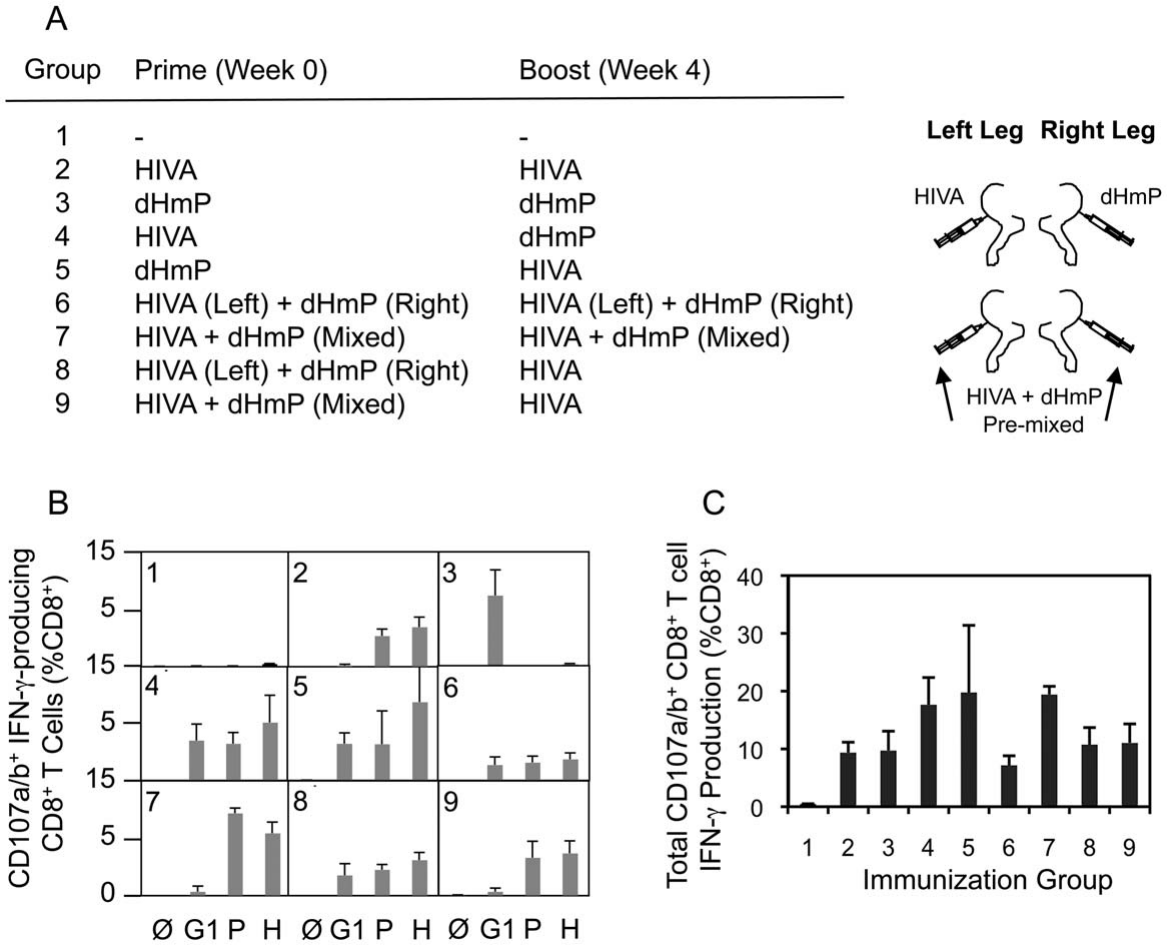
Physical separation of subdominant and dominant epitopes induces broad CD8^+^ T cell responses. A) Groups of 5 BALB/c mice were given 2x a total of 100 µg DNA at 4-wk intervals as indicated in with the HIVA or HIVAdH vaccines being given either mixed or separately into the muscles of the left and right hind legs. After a 23-wk rest, the mice were briefly re-stimulated with recombinant vaccinia virus WR.HIVA delivered i.p. and their H-, P- and G1-specific responses were analyzed using a multicolour flow cytometry 4 d later. B) Graphs show IFN-γ-producing CD8^+^CD107a/b^+^ cell frequencies following an *in vitro* peptide re-stimulation indicated below. C) The graph gives the total IFN-γ production by CD8^+^CD107a/b^+^ cells summing frequencies of G1-, P- and H-specific CD8^+^ CD107a/b^+^ cells. Results in B and C are shown as mean±SD. One out of at two independent experiments is shown.

Next, we wanted to assess whether responses to subdominant epitopes can be improved to the level of the dominant ones by separating their induction either anatomically or in time from the dominant determinants. Employing the HIVA and dHmP constructs, mice were immunized using various regimens of mixed or split injections depicted in Figure 4A. After a 23-wk rest, the mice were briefly re-stimulated with vaccinia virus strain Western Reserve WR.HIVA, sacrificed and their responses were analyzed using a multicolour flow cytometry (Figure 4, also see Figure S3 for representative dot plots). A number of observations were made. Thus, responses to the G1 epitope were significantly increased relative to those specific for epitopes H and P following both HIVA-dHmP and dHmP-HIVA prime-boost regimens (Fig 4B, groups 4 and 5). As for anatomical separation, split vaccine administration for both priming and boosting yielded equivalent T cell frequencies against all three epitopes (4B, groups 6 and 8), while pre-mixed vaccines injected into 2 sites failed to induce anti-G1 responses (Figure 4B, groups 7 and 9). Finally, physically separated (Figure 4B, group 8), but not mixed (Figure 4B, group 9) priming followed by HIVA only boosting also induced equal responses to all three epitopes. Therefore, either temporal or spatial separation of subdominant from dominant epitopes increases the breadth of the T cell responses.

### Epitope hierarchy is affected by vector regimen and can change with time

**Figure 5:**
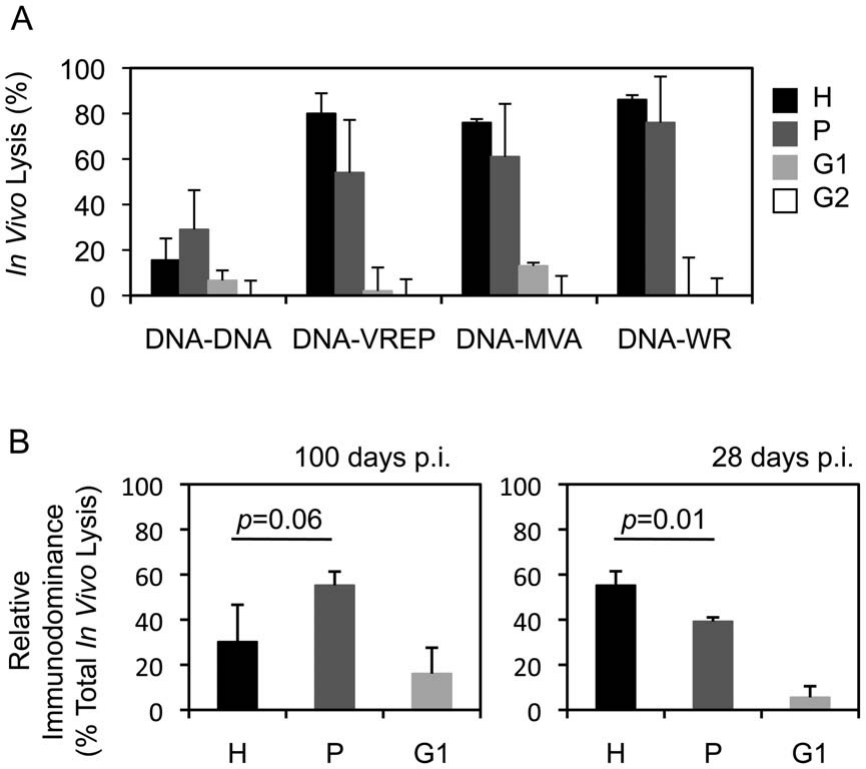
Vector of the boost vaccine influences the epitope hierarchy. A) Groups of 3–4 BALB/c mice were primed with 100 µg of pTHr.HIVA DNA and boosted with the same dose of pTHr.HIVA DNA or 5×10^6^ PFU of MVA.HIVA, VREP.HIVA or WR.HIVA at a 2-wk interval. Hundred d later, cytolytic activity was assessed in an *in vivo* lysis assay using syngeneic peptide-pulsed cells in naïve and vaccinated animals. B) Groups of 3 BALB/c mice received 2x 100 µg of pTHr.HIVA DNA 2 wk apart and assessed for the cytolytic activity at 100 (left) or 28 d (right) post-immunization. The relative immunodominance of responses to individual epitopes was calculated as a percentage of the total CD8^+^ cell response. Results are shown as mean±SD. *p* values for the H and P epitope-specific response comparison are inserted above the columns. Panel B is a repetition of the important observation from panel A. This is further confirmed in Figure 6A.

Vaccine vectors can affect responses to the transgene product by differential activation of innate immunity e.g. through pattern-recognition receptors [39] and by generating CD8^+^ T cell epitopes derived from the vector, which can undesirably interfere or compete with the induction of insert-specific responses [40], [41]. To investigate the influence of vector delivery on the hierarchy of epitopes, groups of BALB/c mice were primed with pTH.HIVA plasmid DNA and boosted with one of four vaccines: pTH.HIVA, MVA.HIVA [24], Semliki Forest Virus replicons VREP.HIVA [42], or a replication-competent vaccinia virus WR.HIVA [20]. Because long-term vaccine protection requires establishment of T cell memory, responses to epitopes H, P, G1 and G2 were measured 100 d after the last vaccination. The most striking difference was observed between the DNA-DNA and the heterologous regimens in that the DNA-DNA-induced H-specific memory T cells no longer dominated the anti-HIVA response and were replaced by T cells specific for peptide P (Figure 5A). This change in the epitope hierarchy over time was confirmed in separate experiments specifically comparing CD8^+^ T cell responses at 100 and 28 d post vaccination (Figure 5B).

### Mutated HIVA vaccines partially protect against vaccinia virus WR.HIVA challenge

**Figure 6:**
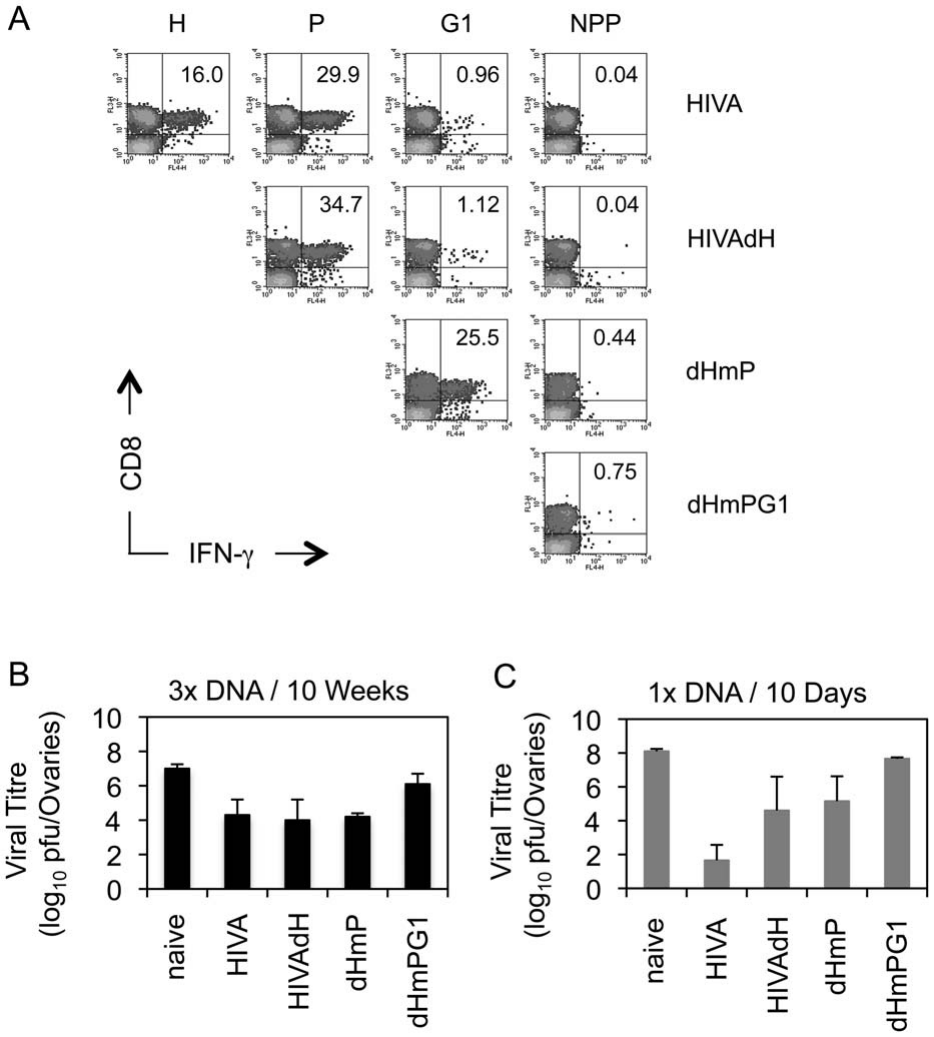
Recovered subdominant epitopes contribute towards protection against vaccinia virus WR.HIVA challenge. A) Groups of 4 BALB/c mice were given 3x 100 µg of DNA expressing the original and deleted forms of the HIVA immunogen at 3-wk intervals. Nine wk later, animals were bled, their PBMC pooled and their vaccine-induced CD8^+^ T cell responses recognizing the H, P, G1 and NPP peptides were assessed by IFN-γ ICS assay. Dot plots are shown with inserted numbers indicating IFN-γ producing cells as a percentage of total CD8^+^ splenocytes. B) Ten wk after the last vaccination, animals were challenged with 2×10^7^ PFU of WR.HIVA i.p., sacrificed 4 d later and the challenge virus titre in ovaries was determined. C) Groups of 3 BALB/c mice were given 1x 100 µg of DNA and challenged with 2×10^7^ PFU of WR.HIVA after 10 d and sacrificed 4 d later for the ovaries titre determination. Results in B and C are shown as mean±SD. Panel A is independently confirmed by Figure 5, and panels B and C show one of two experiments.

Next, protective efficacy of the mutated HIVA immunogens was assessed. Because mice cannot be challenged with HIV-1, replicating vaccinia virus WR.HIVA was used as a surrogate virus challenge. In this model, the WR.HIVA load in ovaries 5 d after challenge serves as the infection quantitative readout [20]. Thus, BALB/c mice were vaccinated 3x with the intact or mutated HIVA immunogens delivered by pTH plasmid DNA. Nine wk after the last vaccination, analysis of the T cell memory induced to the H, P, G1 and NPP determinants in circulating PBMC were carried out prior to the challenge and confirmed shifts in the response hierarchy induced by mutated HIVA immunogens observed above including the loss of the H epitope superiority at this late time point (Figure 6A). One week after the bleed, mice where challenged with WR.HIVA. Consistent with the immunogenicity data, the groups of mice that received the HIVA, HIVAdH and dHmP immunogens showed a similar, approximately 1000-fold decrease in the virus load relative to the naïve group, while the protection induced by dHmPG1 vaccine was 100-fold lower (Figure 6B). To assess the initial dominance of the anti-H response, groups of BALB/c mice were also immunized once with the DNA vaccines and challenged 10 d later. Indeed, early after vaccination, the HIVA immunogen conferred the best protection decreasing the WR.HIVA load by 6 orders of magnitude (Figure 6C). Thus, together the early and late challenge experiments indicated that subdominant epitopes contribute to the protective efficacy and their contribution inversely correlates with their position in the epitope hierarchy: for the HIVA immunogen and WR.HIVA challenge, the stronger response they induce, the bigger their protective role is. Their relative protective efficacy can change with time.

### G1 epitope alone protects against EcoHIV if not dominated

**Figure 7:**
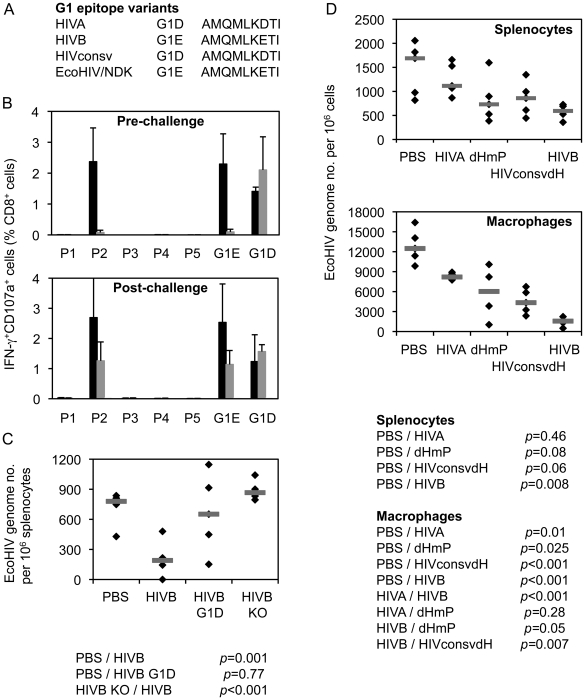
G1 epitope-specific CD8^+^ T cell responses confer protection against EcoHIV/NDK challenge. A) G1 epitope variants in the three used immunogens and the challenge EcoHIV. B) Eight BALB/c mice received 2x 100 µg of DNA of the HIVB vaccine containing epitope G1E (black bars), its G1D variant (dark grey bars) or knock-out versions (KO; light grey bars) and their HIVB-induced responses were determined before (n = 3) or after (n = 5) challenge with EcoHIV/NDK delivered 5 d after the last vaccination. For ICS assay, 5 pools (P1–P5) of overlapping peptides 15/11 across the whole HIVB protein and peptides G1E or G1D were used for re-stimulation as indicated below the graphs. Results are shown as mean±SD. C) The 3 vaccinated and 1 unvaccinated control groups of 5 mice were challenged with EcoHIV/NDK and the EcoHIV/NDK loads in splenocytes were determined using qPCR. The horizontal bars indicate group mean of the genome copy number per 10^6^ splenocytes. D) Mice were immunized as above with plasmid DNA expressing four different immunogens HIVA, dHmP, HIVconsvdH or HIVB containing variants of the G1 epitope or left naïve as indicated below the graphs and challenged with EcoHIV/NDK 5 d later. The EcoHIV/NDK load was determined in the splenocytes and peritoneal macrophages using qPCR. Group means are indicated by horizontal bars.

Protective efficacy against chimaeric EcoHIV/NDK [21], [22] provided by the G1 epitope was assessed when generated from several different immunogens. EcoHIV is particularly suitable for the first, quick assessment of efficacy of T cell-inducing vaccines as it does not express the HIV-1 Env and, therefore, does not carry the H epitope, but contains the clade D version of G1 AMQMLKETI, designated G1E (Figure 7A). Immunogen HIVB is a clade B near-equivalent of HIVA [23], which also contains the G1E epitope, but not the H epitope. In mice immunized twice with pTH.HIVB DNA, both pre- and post-challenge ICS analysis indicated that the G1 epitope generated the only detectable CD8^+^ T cell response (Figure 7B). These G1E-specific responses correlated with lower EcoHIV/NDK load in splenocytes of challenged BALB/c mice (p = 0.001). Amino acid 7E-for-D substitution in the G1 epitope of the HIVB vaccine decreased this protection (p = 0.77) and the epitope knock-out abolished completely vaccine efficacy (Figure 7C). Inferior protection by the G1D epitope was also observed using the HIVA mutant dHmP and our second-generation immunogen HIVconsvdH [28] (Figure 7D). Finally, there was a trend indicating that intact HIVA immunogen was less efficient in inducing protective responses than dHmP presumably because in HIVA, the protective G1D epitope was dominated by irrelevant epitopes H and P (Figure 7D). Thus, G1 when up-ranked as the most immunodominant vaccine epitope is sufficient to induce protective efficacy; this protection may be compromized by irrelevant/non-protective, but superior epitope.

## Discussion

There is a very appealing rationale to focus vaccine-induced HIV-1-specific CD8^+^ T cell responses on invariant determinants of the HIV-1 proteome [12], [28], [29], [43]. In the work presented here, we provide further experimental evidence in support of this approach by demonstrating the protective potential of subdominant epitopes against two surrogate virus challenges. A number of important observations have been made.

First, the pattern of CD8^+^ T cell immunodominance can be altered by modifying vaccine immunogens, therefore, immunodomination is a relative relationship between epitopes rather than an epitope's absolute status. Although the shift in the immunodominance was demonstrated previously for a loss of a single epitope [5], [14], [16], [17], [26], [44], the extent to which this phenomenon applies to other subdominant epitopes within the same protein immunogen of a subunit vaccine has not been investigated. Here, we show that sequential inactivation of three CD8^+^ T cell epitopes on the top of the immunodominance hierarchy leads to a gradual epitope up-ranking reflected in increased magnitudes of the remaining responses.

Second, subdominant epitopes have the potential to replace the stronger ones on the top of the immunodominance hierarchy. This is an important prerequisite for anti-HIV-1 vaccine strategies, which aim to deal with the HIV-1 diversity and escape by directing prophylactic CD8^+^ T cell responses away from the hypervariable regions recognized during primary HIV-1 infection [9] to subdominant, but invariable parts of the HIV-1 proteome [12], [28], [29], [43]. Early focus on the HIV-1 functionally conserved protein sequences, which cannot change without a likely high cost to HIV-1 fitness [45], [46], may provide host immune responses with the critical extra edge in the chase of the ‘ever’-escaping HIV-1 during the primary infection [9], [47]–[50] as well as offer a global universal cross-clade vaccine [28].

Third, we demonstrated that epitope hierarchy can change with time and this change may depend on the means of the immunogen delivery. In the case presented here, the boosting vaccine vector influenced the relative position of epitopes H and P in the order of hierarchy, whereby following a double or triple DNA prime-boost regimen, epitope H was the strongest at 28 d post vaccination, but, by 100 d, was dominated by epitope P. This was not observed for the other investigated boosting vaccine vectors and, thus, the choice of a vaccine vector may have a profound effect on the pattern of dominance within the transgene product. It remains untested whether such hierarchy swap happens only after a single pTH.HIVA DNA delivery. The mechanism for this phenomenon is not known, but it is not likely caused by exhaustion of the H-specific CD8^+^ T cells. We published previously long-term immunogenicity of one pTH.HIVA DNA delivery to BALB/c mice and, although the read out involved only peptide H and not peptide P, 182 days later 51±17 SFU/10^6^ of ex vivo H-specific splenocytes were detected in an IFN-γ ELISPOT assay, and following an additional 5-day-in vitro re-stimulation with H peptide, expanded effectors specifically lysed 25% of ^51^Cr-labeled target cells. In the same experiment, 2x pTH.HIVA DNA induced 89±39 SFU/10^6^ and yielded similar levels of H-specific ^51^Cr release. Thus these cells at least for these two effector functions appeared normal [42]. Other studies investigated response hierarchies to the whole viruses rater than a single protein. For epitopes expressed from poxviruses, shaping of immunodominance during boost, but not prime, was attributed to cross-competition of CD8^+^ T cells [51]. Changes in epitope hierarchy were also reported for other viruses such as lymphocytic choriomeningitis virus (LCMV), influenza virus and herpes viruses and the suspected underlying mechanisms included T cell exhaustion, kinetics of viral protein expression and cross-presentation [4], [52]–[54]. Change with time in epitope hierarchy is well documented for responses to Epstein-Barr virus (EBV) lytic and latent proteins (reviewed in [55]) and shapes the immunodominance of anti-LCMV cytotoxic T cell responses [4]. In both EBV and LCMV infections, this phenomenon was explained by the biology of the virus; in contrast, the H and P epitopes are expressed from the same protein. For the HIVA epitopes, the epitope hierarchy was mirrored by T cell avidity for the MHC-peptide complexes and relative kinetics of individual peptide-specific T cell responses. A degree of competition depending on the affinity of responding T cells was also reported for peptide-pulsed dendritic cells and was much more pronounced for T cells with the same epitope specificity [56], [57]. Thus, a number of mechanisms determine the epitope ranking and their relative importance is likely specific for each antigen and virus/vaccine vector combination studied.

Fourth, generation of a broad CD8^+^ T cell response is a major goal of current vaccine strategies particularly against highly variable infectious agents such as HIV-1 and various ways towards achieving this are suggested in the literature. For example, anatomic separation and heterologous prime-boost regimens partially overcame epitope competition and generated co-dominant responses against two [14] or a number of [29] unequal epitopes derived from HIV-1. Using a string of five human melanoma epitopes, immunodominance could be overcome by boosting of DNA-primed responses using a mixture of recombinant viruses, each encoding different antigenic determinants [58]. Our data suggest that either priming or boosting of responses to subdominant epitopes can be used to increase the response breadth as long as the weaker epitope is allowed to dominate at least once locally or in time. Again, the subtle discrepancies with some published results can be most likely reconciled by differences in individual experimental and/or vaccine designs.

Finally, we demonstrated that subdominant up-ranked epitopes can be protective *in vivo* against viral challenge if a protocol for efficient T cell stimulation by these epitopes can be developed. These results confirm and expand several previous observations [5], [13]–[17] and have implications for design of vaccines inducing CD8^+^ T cell responses in general. However, whether or not a full protective potential of subdominant epitopes can be harnessed by a rational vaccine design also depends on the efficiency of processing and presentation of these subdominant determinants on the surface of HIV-1-infected cells during natural infection. CD4^+^ T cells, the main cells of HIV-1 replication, are not ‘professional primers’ of CD8^+^ T cells (although are likely to expand already primed responses) and many, if not most, T cell responses in natural HIV-1 infection must be initiated by cross-priming [59]. Therefore, detection of responses to subdominant epitopes measured by recognition of peptide-pulsed targets does not guarantee that HIV-1-infected cells express that epitope on their surface. Although escape mutations do occur in conserved regions [60] pointing to an efficient presentation/recognition of at least some epitopes, escape in these regions is less frequent. Indeed, differences between recognition of peptide-pulsed and HIV-1-infected targets were previously reported [61]–[64] and will be the focus of our clinical studies with HIVconsv. Here, we demonstrated that subdominant HIVA epitopes contribute to *in vivo* protection against surrogate WR.HIVA infection and responses to a single subdominant epitope G1 (AMQ) in Gag led to an enhanced clearance of EcoHIV/NDK infection. CD8^+^ T cells specific for a single epitope protected mice against e.g. *Plasmodium berghei* and LCMV [65], [66] and protection after removing dominant epitopes was reported for an experimental infection of rhesus macaques with simian immunodeficiency virus [67]. Without a clear definition of functional correlates of T cell protection against HIV-1 in humans, protection against surrogate virus challenges provides a useful readout for studying the ability of vaccine-elicited responses to control real virus infection *in vivo*. It does remain that these challenge models serve more as experimental tools for studying the workings of the immune system rather than predictors of efficacy of candidate HIV-1 vaccines in humans.

Taken together, the findings reported in this paper provide an important reassurance for vaccine strategies targeting pathogens' subdominant determinants. By the same token, higher-ranked non-protective determinants may over-compete and likely delay T cell induction against potentially protective epitopes. For some natural virus infections, such as by HIV-1, responses to the conserved regions may come too late to win the race with the virus [9].

## Materials and Methods

### Vaccine construction and preparation

Design, construction and preparation of HIVA, HIVB and HIVconsv vaccines were described previously [23], [24], [28]. The epitopes of HIVA (Im,) and HIVB (Hong, D.Phil Thesis, University of Oxford, 2009) genes were deleted or serially mutated using standard techniques of recombinant DNA technology.

### Mice, immunizations, bleeding and preparation of splenocytes

Groups of 5- to 6-wk-old female BALB/c mice were used. Under general anaesthesia, mice were immunized i.m. with indicated doses of plasmid DNA or 5×10^6^ PFU of recombinant MVA (rMVA). Mice were bled using a superficial vein. On the day of sacrifice, spleens and peritoneal macrophages were collected. Splenocytes were isolated by pressing spleens individually through a 70-µM nylon cell strainer (BD Falcon) using a 5-ml syringe rubber plunger. Following the removal of red blood cells with RBC Lysing Buffer Hybri-Max (Sigma), splenocytes were washed and resuspended in R10 for ELISPOT and intracellular cytokine staining (ICS) assays. For in vitro expansion of cells, splenocytes were resuspended in Lymphocyte Medium [R-10 (RPMI 1640 supplemented with 10% FCS, penicillin/ streptomycin), 20 mM HEPES and 15 mM 2-mercaptoethanol] and incubated for 5 d in the presence of peptides or peptide pools (2 µg/ml).

### Ethics statement

All animal procedures and care conformed strictly to the United Kingdom Home Office Guidelines under The Animals (Scientific Procedures) Act 1986. The protocol was approved by the local Research Ethics Committee (Clinical Medicine, University of Oxford). Experiments were carried out under Project Licence no. 30/2406 held by TH with a strict implementation of the Replacement, Reduction and Refinement (3Rs) principles.

### Peptide synthesis

Individual peptides of 9- to 20-mer in length were synthesized in an in-house facility using the Advanced Chemtech automated synthesizer and yielded a purity of >95%. All peptides were dissolved in DMSO (Sigma-Aldrich) to yield a stock of 10 mg/ml, and stored at −80°C.

### T2 peptide binding assay

TAP2 (Transporter Associated Protein 2)-deficient T2 cell lines stably expressing D^d^, K^d^, and L^d^ molecules kindly provided by Dr. Ted Hansen (Washington university, U.S.A.) were used to assess the ability of MHCs to bind each peptide. In brief, 1×10^5^ cells were incubated with 0/0.1/1.0/10/100 µM peptides in no-serum RPMI (R-0) for 14 h at 37°C, 5% CO_2_ in a flat-bottomed 96-well plate. 14 h later, cells were transferred to a round-bottomed 96-well plate and washed with PBS before staining the cells with anti-H-2K^d^/H-2D^d^-PE or anti-H-2L^d^-PE (eBioscience) monoclonal antibodies. Cells were then analyzed on a FACS flow cytometer.

### *In vivo* killing assay

Naïve syngeneic mice were sacrificed, the splenocytes prepared as above and the isolated splenocytes incubated with or without peptides in R-10 at 37°C, 5% CO2 for 90 min and washed 3x. Peptide-unpulsed target cells were labeled with CMTMR (Cell Tracker Orange, Molecular Probes) only, while peptide-pulsed target cells were labeled with CFSE (Molecular Probes) and combined with or without CMTMR as described previously [25]. Differentially labeled cell cultures were washed, resuspended in PBS and combined for intravenous adoptive transfer, with each animal receiving approximately 2×106 cells of each population. After 12 h, animals were sacrificed, and their splenocytes were isolated and analyzed using flow cytometry. Cytotoxicity was calculated using the following formula: Adjusted % survival = 100 x (% survival of peptide-pulsed cells/mean % survival of peptide unpulsed cells), followed by the calculation of % specific lysis = 100 - adjusted % survival [68].

### Intracellular cytokine staining

ICS assay was performed as described previously [25] and stored at 4°C until analysis. Anti-CD107a-FITC/anti-CD107b-FITC (BD Biosciences), anti-CD16/32 (BD Biosciences) at 4°C for 30 minutes. All subsequent antibody stains were performed using the same conditions. Cells were then washed and stained with anti-CD8-PerCP/Pacific Blue, anti-CD4-APC-Cy7 (BD Biosciences), anti-IFN-γ-APC and anti-TNF-α-PE (BD Biosciences) mAbs were used.

### IFN-γ ELISPOT assay

The ELISPOT assay was performed using the Mouse IFN-γ ELISpot kit (Mabtech) according to the manufacturer's instructions. Spots were visualised using sequential applications of a biotin-conjugated secondary anti-IFN-**γ** mAb (R4-6A2, Rat IgG1), an alkaline phosphatase and a chromogenic substrate (Bio-Rad) and counted using the AID ELISpot Reader System (Autoimmun Diagnostika).

### Vaccinia virus WR.HIVA challenge/protection assay

Groups of BALB/c female mice naïve or immunized were challenged with 2×10^6^ PFU of vaccinia virus WR.HIVA i.p. Four d later, ovaries were collected and homogenized using MagNA Lyser Green Beads (Roche) in a bead-based homogenizer. Confluent Hu-TK^-^143B cells in 6-well plates were infected in duplicates with 10-fold serial dilutions of the homogenized ovaries. Cells were stained with 0.1% crystal violet in 20% ethanol and the numbers of plaques were counted.

### FACS analysis

All chromogen-labeled cells were acquired using FACSCalibur and CyAn ADP (Dako) flow cytometer and analyzed with CellQuest software (BD Biosciences) and Flowjo (Tree Star), respectively.

### Preparation of EcoHIV/NDK stock and challenge

EcoHIV/NDK stocks were prepared as described previously [22]. Briefly, plasmid DNA containing the infectious viral genome was transfected into 293T cells, the virus was harvested from the culture supernatant after two sequential 1-d incubations, concentrated by centrifugation and quantified by determination of the Gag p24 content (Zeptometrix Corporation, Buffalo, NY) and stored at −80°C until use. Mice were challenge with 2 µg p24 cell-free virus by i.p. injection.

### Quantitative real-time PCR (qPCR)

DNA from splenocytes and macrophages was isolated on the day of euthanasia and qPCR was conducted using primers sense: 5′-FTTAGCACTTGCTTGGGACGA, antisense: 5′-TGTCCCAGAAGTTCCACART, Doubledye Taqman probe 5′-TWGCACTTWTCTGGGACGA (F = G or C; R = A or G; W = A or T) and ABI Prism 7700 instrument. Data are reported as numbers of viral DNA copies per 10^6^ of cells, cell numbers were obtained by qPCR amplification of normalizing gene using gDNA kit. Custom primers and probes detecting challenge virus DNA, but not vaccines, and the house keeping gene were designed and optimized by PrimerDesign (Southampton, UK).

### Statistical analysis

The *p* values associated with an unpaired Student's t-Test with a two-tailed distribution were determined for differences between both frequencies of vaccine-induced HIV-1-specific T cells and EcoHIV/NDK genome copy numbers following challenge.

## Supporting Information

Figure S1 Stability and expression of modified immunogens. A) Stability of proteins HIVA (top) and dHmP (bottom) were analysed using [35S]Methionine pulse-chase experiment. Briefly, 293T cells were incubated with [35S]Methionine for 16 h post transfection and chased for 0, 2, 4, 8 and 24 h using unlabeled medium. Cells were lysed and the recombinant proteins were immunoprecipitated using anti-Pk antibody and separated on SDS-PAGE. Following overnight exposure, bands were quantifies using BioSpectrum Imaging System as depicted on the right. B) Western blot analysis of 293T cells transiently transformed with plasmid pTH expressing HIVB (lane 1), HIVB-G1D (lane 2), HIVB-KO (lane 3) or empty pTH. Proteins in cell lysates were separated using SDSPAGE and the recombinant proteins were detected via the C-terminal Pk tag utilizing anti- Pk mAb and HRP-conjugated protein A followed by ECL.
(PDF)

Figure S2 T2 assays for peptide binding affinity for MHC class I. TAPdeficient T2 cell lines stably transformed with H-2Dd, H-2Kd or H-2Ld complexes were kindly provided by Dr Hansen, Washington University. Peptide-loaded MHC complexes were detected by fluorochrome-conjugated mAb (DB Biosciences) and the cells were analyzed using flow cytometer. A) Index and mutated peptides P and G1 or B) peptide NNP as indicated above the histograms as peptide-MHC were tested at decreasing concentrations ranging from 100 µM to 0.01 µM (pink 100 µM, turquoise 10 µM, orange 1 µM, green 0.1 µM, blue 0.01 µM and grey filled no peptide) for binding to MHC class I complexes.
(PDF)

Figure S3 Spatial and temporal separation of dominant and subdominant epitopes during vaccination (Additional data for Figure 4). Groups of 4 BALB/c mice were given 2x a total of 100 µg DNA at 4-wk intervals as indicated in Figure 4B with the HIVA or HIVAdH vaccines being given either mixed or separately into the muscles of the left and right hind legs. After a 23-wk rest, the mice were briefly re-stimulated with recombinant vaccinia virus WR.HIVA delivered i.p. and their H, P and G1-specific responses were analyzed using a multicolour flow cytometry 4 d later. Representative examples are shown from one mouse of each group.
(PDF)

## References

[1] Yewdell JW (2006). Confronting complexity: real-world immunodominance in antiviral CD8+ T cell responses.. Immunity.

[2] Brehm MA, Pinto AK, Daniels KA, Schneck JP, Welsh RM (2002). T cell immunodominance and maintenance of memory regulated by unexpectedly cross-reactive pathogens.. Nat Immunol.

[3] Mo AX, van Lelyveld SF, Craiu A, Rock KL (2000). Sequences that flank subdominant and cryptic epitopes influence the proteolytic generation of MHC class I-presented peptides.. J Immunol.

[4] Probst HC, Tschannen K, Gallimore A, Martinic M, Basler M (2003). Immunodominance of an antiviral cytotoxic T cell response is shaped by the kinetics of viral protein expression.. J Immunol.

[5] van der Most RG, Murali-Krishna K, Lanier JG, Wherry EJ, Puglielli MT (2003). Changing immunodominance patterns in antiviral CD8 T-cell responses after loss of epitope presentation or chronic antigenic stimulation.. Virology.

[6] Yewdell JW, Bennink JR (1999). Immunodominance in major histocompatibility complex class I-restricted T lymphocyte responses.. Annu Rev Immunol.

[7] Gaschen B, Taylor J, Yusim K, Foley B, Gao F (2002). Diversity considerations in HIV-1 vaccine selection.. Science.

[8] Barouch DH, Kunstman J, Kuroda MJ, Schmitz JE, Santra S (2002). Eventual AIDS vaccine failure in a rhesus monkey by viral escape from cytotoxic T lymphocytes.. Nature.

[9] Goonetilleke N, Liu MK, Salazar-Gonzalez JF, Ferrari G, Giorgi E (2009). The first T cell response to transmitted/founder virus contributes to the control of acute viremia in HIV-1 infection.. J Exp Med.

[10] Goulder PJ, Watkins DI (2004). HIV and SIV CTL escape: implications for vaccine design.. Nat Rev Immunol.

[11] Moore CB, John M, James IR, Christiansen FT, Witt CS (2002). Evidence of HIV-1 adaptation to HLA-restricted immune responses at a population level.. Science.

[12] Frahm N, Kiepiela P, Adams S, Linde CH, Hewitt HS (2006). Control of human immunodeficiency virus replication by cytotoxic T lymphocytes targeting subdominant epitopes.. Nat Immunol.

[13] Gallimore A, Dumrese T, Hengartner H, Zinkernagel RM, Rammensee HG (1998). Protective immunity does not correlate with the hierarchy of virus-specific cytotoxic T cell responses to naturally processed peptides.. J Exp Med.

[14] Liu J, Ewald BA, Lynch DM, Nanda A, Sumida SM (2006). Modulation of DNA vaccine-elicited CD8+ T-lymphocyte epitope immunodominance hierarchies.. J Virol.

[15] Rodriguez F, Slifka MK, Harkins S, Whitton JL (2001). Two overlapping subdominant epitopes identified by DNA immunization induce protective CD8(+) T-cell populations with differing cytolytic activities.. J Virol.

[16] van der Most RG, Concepcion RJ, Oseroff C, Alexander J, Southwood S (1997). Uncovering subdominant cytotoxic T-lymphocyte responses in lymphocytic choriomeningitis virus-infected BALB/c mice.. J Virol.

[17] von Herrath MG, Dockter J, Nerenberg M, Gairin JE, Oldstone MB (1994). Thymic selection and adaptability of cytotoxic T lymphocyte responses in transgenic mice expressing a viral protein in the thymus.. J Exp Med.

[18] McMichael AJ, Hanke T (2003). HIV vaccines 1983-2003.. Nat Med.

[19] Bridgeman A, Roshorm Y, Lockett LJ, Xu ZZ, Hopkins R (2010). Ovine atadenovirus, a novel and highly immunogenic vector in prime-boost studies of a candidate HIV-1 vaccine.. Vaccine.

[20] Im EJ, Saubi N, Virgili G, Sander C, Teoh D (2007). Vaccine platform for prevention of tuberculosis and mother-to-child transmission of human immunodeficiency virus type 1 through breastfeeding.. J Virol.

[21] Nitkiewicz J, Chao W, Bentsman G, Li J, Kim SY (2004). Productive infection of primary murine astrocytes, lymphocytes, and macrophages by human immunodeficiency virus type 1 in culture.. J Neurovirol.

[22] Potash MJ, Chao W, Bentsman G, Paris N, Saini M (2005). A mouse model for study of systemic HIV-1 infection, antiviral immune responses, and neuroinvasiveness.. Proc Natl Acad Sci U S A.

[23] Roshorm Y, Hong JP, Kobayashi N, McMichael AJ, Volsky DJ (2009). Novel HIV-1 clade B candidate vaccines designed for HLA-B*5101^+^ patients protected mice against chimaeric EcoHIV challenge.. Eur J Immunol.

[24] Hanke T, McMichael AJ (2000). Design and construction of an experimental HIV-1 vaccine for a year-2000 clinical trial in Kenya.. Nat Med.

[25] Im EJ, Hanke T (2007). Short communication: preclinical evaluation of candidate HIV type 1 vaccines in inbred strains and an outbred stock of mice.. AIDS Res Hum Retroviruses.

[26] Larke N, Im E-J, Wagner R, Williamson C, Williamson A-L (2007). Combined single-clade candidate HIV-1 vaccines induce T cell responses limited by multiple forms of *in vivo* immune interference.. Eur J Immunol.

[27] Liu Y, McNevin J, Rolland M, Zhao H, Deng W (2009). Conserved HIV-1 epitopes continuously elicit subdominant cytotoxic T-lymphocyte responses.. J Infect Dis.

[28] Letourneau S, Im E-J, Mashishi T, Brereton C, Bridgeman A (2007). Design and pre-clinical evaluation of a universal HIV-1 vaccine.. PLoS ONE.

[29] Rosario M, Bridgeman A, Quakkelaar ED, Quigley MF, Hill BJ (2010). Long peptides induce polyfunctional T cells against conserved regions of HIV-1 with superior breadth to single-gene vaccines in macaques.. Eur J Immunol.

[30] Hanke T, Goonetilleke N, McMichael AJ, Dorrell L (2007). Clinical experience with plasmid DNA- and modified vaccinia vaccine Ankara (MVA)-vectored HIV-1 clade A vaccine inducing T cells.. J Gen Virol.

[31] Takahashi H, Cohen J, Hosmalin A, Cease KB, Houghten R (1988). An immunodominant epitope of the human immunodeficiency virus envelope glycoprotein gp160 recognized by class I major histocompatibility molecule-restricted murine cytotoxic T lymphocytes.. Proc Natl Acad Sci USA.

[32] Hanke T, Schneider J, Gilbert SC, Hill AVS, McMichael A (1998). DNA multi-CTL epitope vaccines for HIV and *Plasmodium falciparum*: Immunogenicity in mice.. Vaccine.

[33] Nakagawa Y, Kikuchi H, Takahashi H (2007). Molecular analysis of TCR and peptide/MHC interaction using P18-I10-derived peptides with a single D-amino acid substitution.. Biophys J.

[34] Mata M, Travers PJ, Liu Q, Frankel FR, Paterson Y (1998). The MHC class I-restricted immune response to HIV-gag in BALB/c mice selects a single epitope that does not have a predictable MHC-binding motif and binds to Kd through interactions between a glutamine at P3 and pocket D. J Immunol.

[35] Didierlaurent A, Ramirez JC, Gherardi M, Zimmerli SC, Graf M (2004). Attenuated poxviruses expressing a synthetic HIV protein stimulate HLA-A2-restricted cytotoxic T-cell responses.. Vaccine.

[36] Mwau M, McMichael AJ, Hanke T (2002). Design and Validation of an ELISPOT Assay for Use in Clinical Trials of Candidate HIV Vaccines.. AIDS Res Hum Retroviruses.

[37] Shastri N, Serwold T, Gonzalez F (1995). Presentation of endogenous peptide/MHC class I complexes is profoundly influenced by specific C-terminal flanking residues.. J Immunol.

[38] Betts MR, Brenchley JM, Price DA, De Rosa SC, Douek DC (2003). Sensitive and viable identification of antigen-specific CD8+ T cells by a flow cytometric assay for degranulation.. J Immunol Methods.

[39] Schulz O, Diebold SS, Chen M, Naslund TI, Nolte MA (2005). Toll-like receptor 3 promotes cross-priming to virus-infected cells.. Nature.

[40] Harrington LE, Most Rv R, Whitton JL, Ahmed R (2002). Recombinant vaccinia virus-induced T-cell immunity: quantitation of the response to the virus vector and the foreign epitope.. J Virol.

[41] Tscharke DC, Woo WP, Sakala IG, Sidney J, Sette A (2006). Poxvirus CD8+ T-cell determinants and cross-reactivity in BALB/c mice.. J Virol.

[42] Hanke T, Barnfield C, Wee EG-T, Ågren L, Samuel RV (2003). Construction and immunogenicity in a prime-boost regimen of a Semliki Forest virus-vectored experimental HIV clade A vaccine.. J Gen Virol.

[43] Rolland M, Nickle DC, Mullins JI (2007). HIV-1 group M conserved elements vaccine.. PLoS Pathog.

[44] Zajac AJ, Blattman JN, Murali-Krishna K, Sourdive DJ, Suresh M (1998). Viral Immune evasion due to persistence of activated T cells without effector function.. J Exp Med.

[45] Altfeld M, Addo MM, Rosenberg ES, Hecht FM, Lee PK (2003). Influence of HLA-B57 on clinical presentation and viral control during acute HIV-1 infection.. AIDS.

[46] Kelleher AD, Long C, Holmes EC, Allen RL, Wilson J (2001). Clustered mutations in HIV-1 gag are consistently required for escape from HLA-B27-restricted cytotoxic T lymphocyte responses.. J Exp Med.

[47] Goulder PJ, Altfeld MA, Rosenberg ES, Nguyen T, Tang Y (2001). Substantial differences in specificity of HIV-specific cytotoxic T cells in acute and chronic HIV infection.. J Exp Med.

[48] McMichael AJ, Borrow P, Tomaras GD, Goonetilleke N, Haynes BF (2010). The immune response during acute HIV-1 infection: clues for vaccine development.. Nat Rev Immunol.

[49] Price DA, Goulder PJ, Klenerman P, Sewell AK, Easterbrook PJ (1997). Positive selection of HIV-1 cytotoxic T lymphocyte escape variants during primary infection.. Proc Natl Acad Sci USA.

[50] Walker BD, Goulder PJ (2000). AIDS. Escape from the immune system.. Nature.

[51] Kastenmuller W, Gasteiger G, Gronau JH, Baier R, Ljapoci R (2007). Cross-competition of CD8+ T cells shapes the immunodominance hierarchy during boost vaccination.. J Exp Med.

[52] Belz GT, Xie W, Altman JD, Doherty PC (2000). A previously unrecognized H-2D(b)-restricted peptide prominent in the primary influenza A virus-specific CD8(+) T-cell response is much less apparent following secondary challenge.. J Virol.

[53] Chen W, Pang K, Masterman KA, Kennedy G, Basta S (2004). Reversal in the immunodominance hierarchy in secondary CD8+ T cell responses to influenza A virus: roles for cross-presentation and lysis-independent immunodomination.. J Immunol.

[54] Nugent CT, McNally JM, Chervenak R, Wolcott RM, Jennings SR (1995). Differences in the recognition of CTL epitopes during primary and secondary responses to herpes simplex virus infection in vivo.. Cell Immunol.

[55] Hislop AD, Taylor GS, Sauce D, Rickinson AB (2007). Cellular responses to viral infection in humans: Lessons from Epstein-Barr virus.. Annu Rev Immunol.

[56] Kedl RM, Schaefer BC, Kappler JW, Marrack P (2002). T cells down-modulate peptide-MHC complexes on APCs in vivo.. Nat Immunol.

[57] Willis RA, Kappler JW, Marrack PC (2006). CD8 T cell competition for dendritic cells in vivo is an early event in activation.. Proc Natl Acad Sci U S A.

[58] Palmowski MJ, Choi EM, Hermans IF, Gilbert SC, Chen JL (2002). Competition between CTL narrows the immune response induced by prime-boost vaccination protocols.. J Immunol.

[59] Yewdell JW (2010). Designing CD8+ T cell vaccines: it's not rocket science (yet).. Curr Opin Immunol.

[60] Leslie AJ, Pfafferott KJ, Chetty P, Draenert R, Addo MM (2004). HIV evolution: CTL escape mutation and reversion after transmission.. Nat Med.

[61] Bennett MS, Ng HL, Ali A, Yang OO (2008). Cross-clade detection of HIV-1-specific cytotoxic T lymphocytes does not reflect cross-clade antiviral activity.. J Infect Dis.

[62] D'Souza MP, Altfeld M (2008). Measuring HIV-1-specific T cell immunity: how valid are current assays?. J Infect Dis.

[63] Le Gall S, Stamegna P, Walker BD (2007). Portable flanking sequences modulate CTL epitope processing.. J Clin Invest.

[64] Tenzer S, Wee E, Burgevin A, Stewart-Jones G, Friis L (2009). Antigen processing influences HIV-specific cytotoxic T lymphocyte immunodominance.. Nat Immunol.

[65] Schneider J, Gilbert SC, Blanchard TJ, Hanke T, Robson KJ (1998). Enhanced immunogenicity for CD8^+^ T cell induction and complete protective efficacy of malaria DNA vaccination by boosting with modified vaccinia virus Ankara.. Nature Med.

[66] Sedlik C, Dadaglio G, Saron MF, Deriaud E, Rojas M (2000). In vivo induction of a high-avidity, high-frequency cytotoxic T-lymphocyte response is associated with antiviral protective immunity.. J Virol.

[67] Valentine LE, Watkins DI (2008). Relevance of studying T cell responses in SIV-infected rhesus macaques.. Trends Microbiol.

[68] Hermans IF, Silk JD, Yang J, Palmowski MJ, Gileadi U (2004). The VITAL assay: a versatile fluorometric technique for assessing CTL- and NKT-mediated cytotoxicity against multiple targets in vitro and in vivo.. J Immunol Methods.

