# Supplementary material for: Protective Efficacy of Serially Up-Ranked Subdominant CD8+ T Cell Epitopes against Virus Challenges
Source: PLoS Pathog. 2011 May 19;7(5):e1002041. doi: 10.1371/journal.ppat.1002041 (PMC3098219; doi:10.1371/journal.ppat.1002041)
Supplement: Figure S1 — Stability and expression of modified immunogens. A) Stability of proteins HIVA (top) and dHmP (bottom) were analysed using [35S]Methionine pulse-chase experiment. Briefly, 293T cells were incubated with [35S]Methionine for 16 h post transfection and chased for 0, 2, 4, 8 and 24 h using unlabeled medium. Cells were lysed and the recombinant proteins were immunoprecipitated using anti-Pk antibody and separated on SDS-PAGE. Following overnight exposure, bands were quantifies using BioSpectrum Imaging System as depicted on the right. B) Western blot analysis of 293T cells transiently transformed with plasmid pTH expressing HIVB (lane 1), HIVB-G1D (lane 2), HIVB-KO (lane 3) or empty pTH. Proteins in cell lysates were separated using SDSPAGE and the recombinant proteins were detected via the C-terminal Pk tag utilizing anti- Pk mAb and HRP-conjugated protein A followed by ECL. (PDF) [file ppat.1002041.s001.pdf]

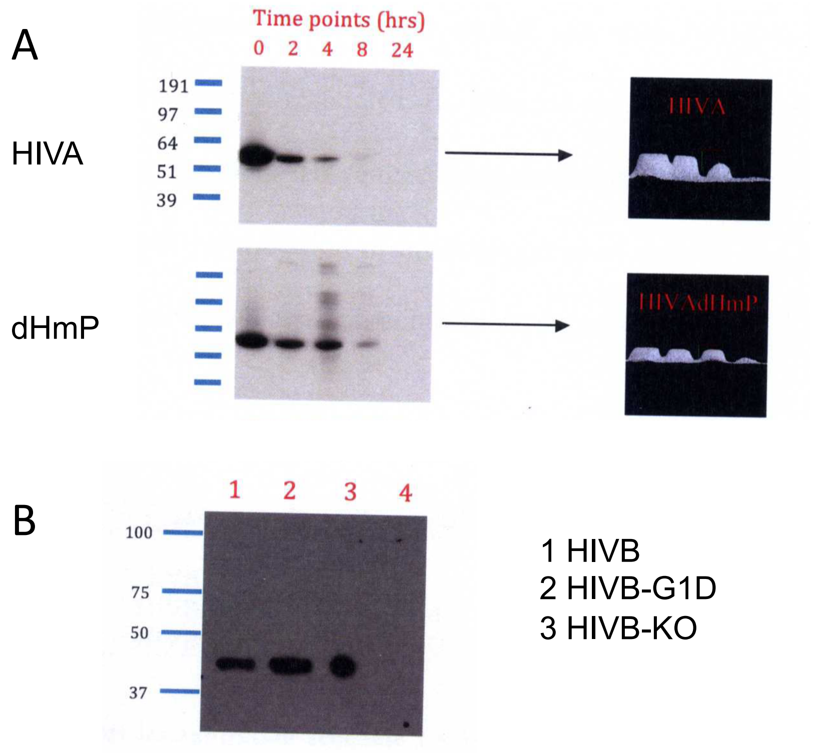

Supplementary Figure S1. Stability and expression of modified immunogens. A) Stability of proteins HIVA (top) and dHmP (bottom) were analysed using [^{35}S]Methionine pulse-chase experiment. Briefly, 293T cells were incubated with [^{35}S]Methionine for 16 h post transfection and chased for 0, 2, 4, 8 and 24 h using unlabeled medium. Cells were lysed and the recombinant proteins were immunoprecipitated using anti-Pk antibody and separated on SDS-PAGE. Following overnight exposure, bands were quantifies using BioSpectrum Imaging System as depicted on the right. B) Western blot analysis of 293T cells transiently transformed with plasmid pTH expressing HIVB (lane 1), HIVB-G1D (lane 2), HIVB-KO (lane 3) or empty pTH. Proteins in cell lysates were separated using SDS-PAGE and the recombinant proteins were detected via the C-terminal Pk tag utilizing anti-Pk mAb and HRP-conjugated protein A followed by ECL.
