# Supplementary material for: Protective Efficacy of Serially Up-Ranked Subdominant CD8+ T Cell Epitopes against Virus Challenges
Source: PLoS Pathog. 2011 May 19;7(5):e1002041. doi: 10.1371/journal.ppat.1002041 (PMC3098219; doi:10.1371/journal.ppat.1002041)
Supplement: Figure S2 — T2 assays for peptide binding affinity for MHC class I. TAPdeficient T2 cell lines stably transformed with H-2Dd, H-2Kd or H-2Ld complexes were kindly provided by Dr Hansen, Washington University. Peptide-loaded MHC complexes were detected by fluorochrome-conjugated mAb (DB Biosciences) and the cells were analyzed using flow cytometer. A) Index and mutated peptides P and G1 or B) peptide NNP as indicated above the histograms as peptide-MHC were tested at decreasing concentrations ranging from 100 µM to 0.01 µM (pink 100 µM, turquoise 10 µM, orange 1 µM, green 0.1 µM, blue 0.01 µM and grey filled no peptide) for binding to MHC class I complexes. (PDF) [file ppat.1002041.s002.pdf]

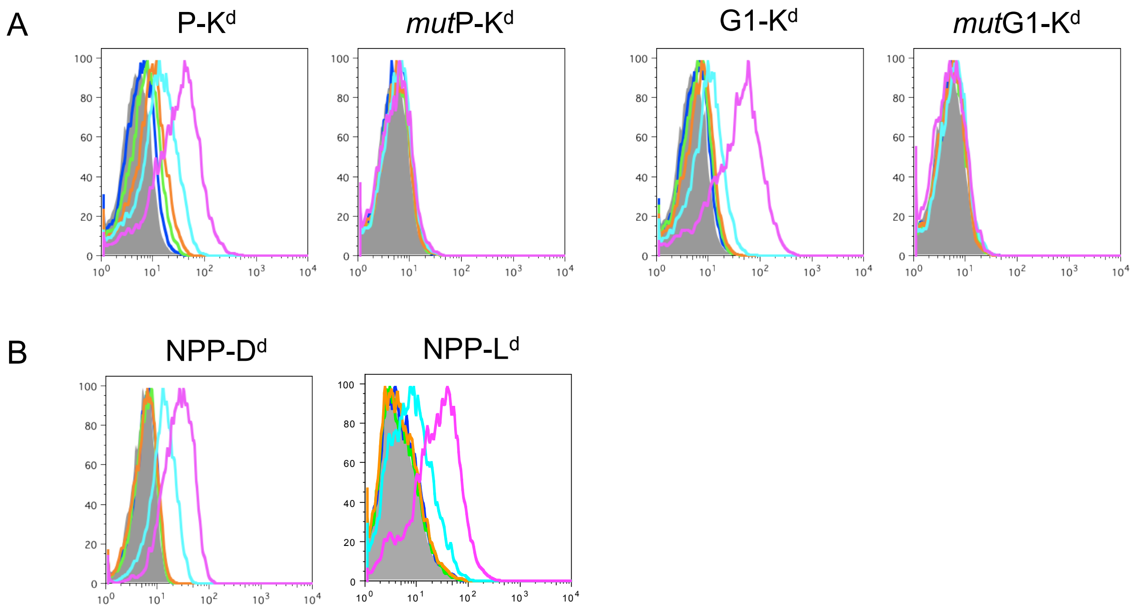

Supplementary Figure S2. T2 assays for peptide binding affinity for MHC class I. TAP-deficient T2 cell lines stably transformed with H-2D^d, H-2K^d or H-2L^d complexes were kindly provided by Dr Hansen, Washington University. Peptide-loaded MHC complexes were detected by fluorochrome-conjugated mAb (DB Biosciences) and the cells were analyzed using flow cytometer. A) Index and mutated peptides P and G1 or B) peptide NNP as indicated above the histograms as peptide-MHC were tested at decreasing concentrations ranging from 100 μM to 0.01 μM (pink 100 μM, turquoise 10 μM, orange 1 μM, green 0.1 μM, blue 0.01 μM and grey filled no peptide) for binding to MHC class I complexes.
