# Supplementary material for: Protective Efficacy of Serially Up-Ranked Subdominant CD8+ T Cell Epitopes against Virus Challenges
Source: PLoS Pathog. 2011 May 19;7(5):e1002041. doi: 10.1371/journal.ppat.1002041 (PMC3098219; doi:10.1371/journal.ppat.1002041)
Supplement: Figure S3 — Spatial and temporal separation of dominant and subdominant epitopes during vaccination (Additional data for Figure 4). Groups of 4 BALB/c mice were given 2x a total of 100 µg DNA at 4-wk intervals as indicated in Figure 4B with the HIVA or HIVAdH vaccines being given either mixed or separately into the muscles of the left and right hind legs. After a 23-wk rest, the mice were briefly re-stimulated with recombinant vaccinia virus WR.HIVA delivered i.p. and their H, P and G1-specific responses were analyzed using a multicolour flow cytometry 4 d later. Representative examples are shown from one mouse of each group. (PDF) [file ppat.1002041.s003.pdf]

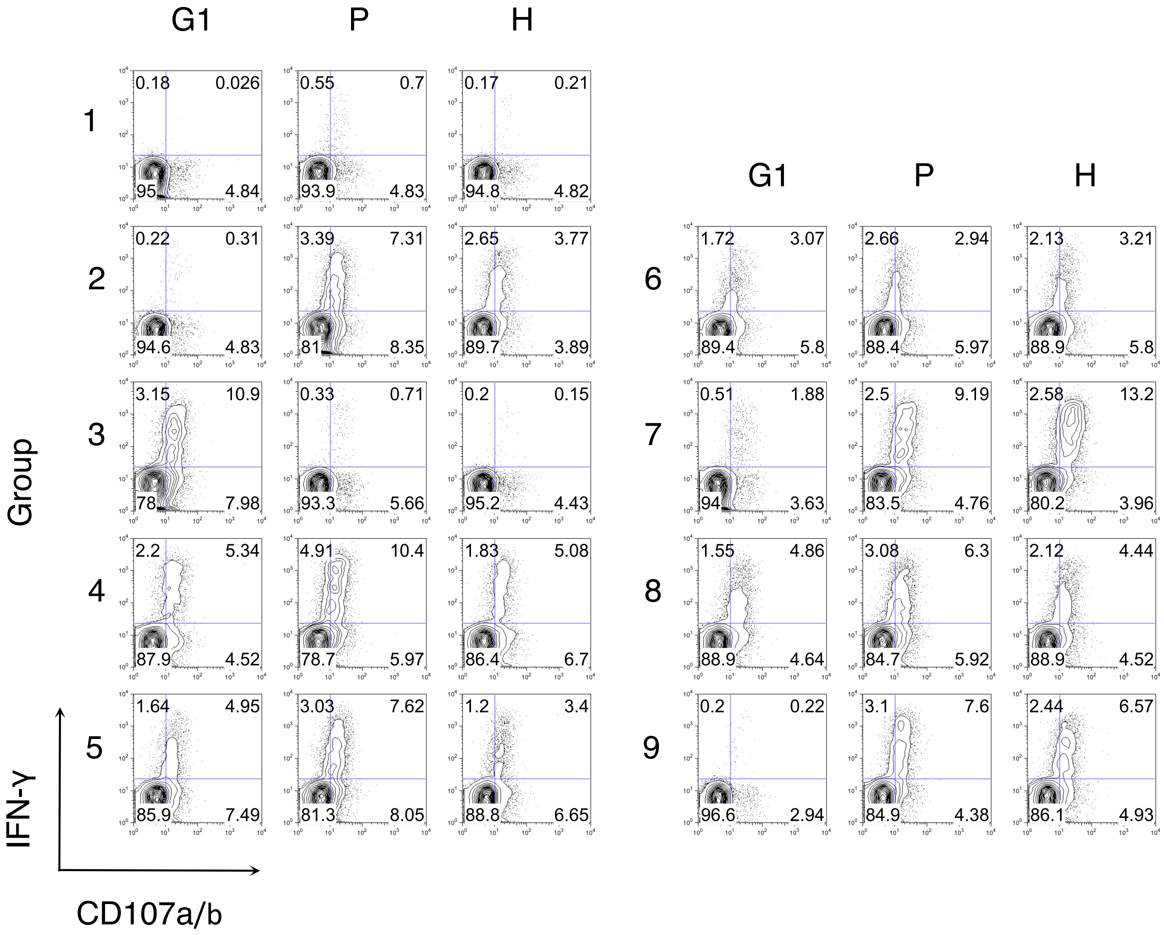

Supplementary Figure S3. Spatial and temporal separation of dominant and subdominant epitopes during vaccination (Additional data for Figure 4). Groups of 4 BALB/c mice were given 2x a total of 100 μg DNA at 4-wk intervals as indicated in Figure 4B with the HIVA or HIVAdH vaccines being given either mixed or separately into the muscles of the left and right hind legs. After a 23-wk rest, the mice were briefly re-stimulated with recombinant vaccinia virus WR.HIVA delivered i.p. and their H, P and G1-specific responses were analyzed using a multicolour flow cytometry 4 d later. Representative examples are shown from one mouse of each group.
